# Molecular Genotyping of *Anisakis* Larvae in Middle Eastern Japan and Endoscopic Evidence for Preferential Penetration of Normal over Atrophic Mucosa

**DOI:** 10.1371/journal.pone.0089188

**Published:** 2014-02-28

**Authors:** Toshio Arai, Nobuaki Akao, Takenori Seki, Takashi Kumagai, Hirofumi Ishikawa, Nobuo Ohta, Nobuto Hirata, So Nakaji, Kenji Yamauchi, Mitsuru Hirai, Toshiyasu Shiratori, Masayoshi Kobayashi, Hiroyuki Fujii, Eiji Ishii, Mikio Naito, Shin-ichi Saitoh, Toshikazu Yamaguchi, Nobumitsu Shibata, Masamune Shimo, Toshihiro Tokiwa

**Affiliations:** 1 Department of Environmental Parasitology, Graduate School of Tokyo Medical and Dental University, Bunkyo-ku, Tokyo, Japan; 2 Department of Gastroenterology, Kameda Medical Center, Kamogawa, Chiba, Japan; 3 Department of Endoscopy, Kameda Medical Center, Kamogawa, Chiba, Japan; 4 Department of Gastroenterology, Toukatsu Hospital, Nagareyama-shi, Chiba, Japan; 5 Department of Pathology, Toukatsu Hospital, Nagareyama-shi, Chiba, Japan; 6 Laboratory of Veterinary Pathology, Azabu University, Sagamihara, Kanagawa, Japan; National Cancer Center, Japan

## Abstract

**Background:**

Anisakiasis is a parasitic disease caused primarily by *Anisakis* spp. larvae in Asia and in Western countries. The aim of this study was to investigate the genotype of *Anisakis* larvae endoscopically removed from Middle Eastern Japanese patients and to determine whether mucosal atrophy affects the risk of penetration in gastric anisakiasis.

**Methods:**

In this study, 57 larvae collected from 44 patients with anisakiasis (42 gastric and 2 colonic anisakiasis) were analyzed retrospectively. Genotyping was confirmed by restriction fragment length polymorphism (RFLP) analysis of ITS regions and by sequencing the mitochondrial small subunit (SSU) region. In the cases of gastric anisakiasis, correlation analyses were conducted between the frequency of larval penetration in normal/atrophic area and the manifestation of clinical symptoms.

**Results:**

Nearly all larvae were *A. simplex* seusu stricto (s.s.) (99%), and one larva displayed a hybrid genotype. The *A. simplex* larvae penetrated normal mucosa more frequently than atrophic area (p = 0.005). Finally, patients with normal mucosa infection were more likely to exhibit clinical symptoms than those with atrophic mucosa infection (odds ratio, 6.96; 95% confidence interval, 1.52–31.8).

**Conclusions:**

In Japan, *A. simplex* s.s. is the main etiological agent of human anisakiasis and tends to penetrate normal gastric mucosa. Careful endoscopic examination of normal gastric mucosa, particularly in the greater curvature of the stomach will improve the detection of *Anisakis* larvae.

## Introduction

Anisakiasis is a parasitic infection caused by nematodes, particularly *Anisakis simplex*, *A. physeteris* or *Pseudoterranova decipiens* [1]. A total of 2,000 cases of Anisakiasis are reported every year worldwide, with more than 90% of the cases in Japan. The disease is contracted by eating raw or undercooked fish contaminated with the parasites. The first cases of anisakiasis were reported in the Netherlands, followed by widespread infestation in Japan [2]. In the past decade, the incidence of anisakiasis has been increasing in several countries, including Austria and Italy [1]–[5]. Most patients are infected by *A. simplex* worldwide. Isolated cases of *A. physeteris* infection were identified in Japan and Spain [1], [5]. Pseudoterranovasis has been reported primarily in the United States and Canada, with a few reports in south Japan and Europe [1], [5]–[7].

Recent molecular studies using polymerase chain reaction-restriction fragment length polymorphism (PCR-RFLP) analysis and sequencing of the internal transcribed spacer (ITS) region revealed that *A. simplex* consists of 3 major sibling species: *A. simplex* seusu stricto (s.s.), *A. pegreffii*, and *A. simplex* C [8]–[10]. In North and South Japan, *A. simplex* s.s. is the most common species. However, the source of infection has not been identified, and no study has investigated regional differences in *Anisakis* spp. in Central Japan [10].

Currently, the only effective treatment is the endoscopic removal of the live larvae from the gastric mucosa. Therefore, it is important to find the larvae quickly and remove them thoroughly. Only few studies examined endoscopic findings of gastric anisakiasis [11], [12]. The *Anisakis* larvae are usually found in the greater curvature [11]. However, they are often hidden in the gastric folds. Furthermore, the mucosa undergoes a series of morphological changes from the time of contaminated food ingestion to the endoscopic examination, including edema and tumor formation, which may hide the larvae [12]. Furthermore, it is often hidden among the gastric folds with mucosal edema. For these reasons, the endoscopic detection and removal of all live larvae remains a challenge, and more information is required on the penetration sites.

To the best of our knowledge, no study has investigated whether the health status of the mucosa affects *Anisakis* penetration in the human stomach. A recent *in vitro* study showed that *Anisakis* penetrates agar gel more easily under acidic conditions [13]. In the stomach, the pH of normal mucosa is lower than that of atrophic area [14]. Therefore, we hypothesized that *Anisakis* may preferentially invade normal gastric mucosa. In this report, we genotyped *Anisakis* larvae removed from patients in Middle Eastern part of Japan and tested the impact of mucosal properties on larval penetration.

## Materials and Methods

### Patients

From January 2011 to December 2012, 44 patients with anisakiasis visited three hospitals of Middle Eastern Japan (Kameda General Hospital, Kameda Clinic and Toukatsu Hospital). A total of 55 larvae were removed by upper gastrointestinal endoscopic examination and 2 larvae were removed by colonoscopy.

### Morphological examination

The collected larvae were sent to our laboratory and morphological observation was carried out under a light microscope. We identified the genus and stage of the larvae, as previously described [6], [15], [16].

### Molecular identification

Total DNA was extracted with the Get *pure* DNA Kit-Cell, Tissue (Dojindo, Japan) according to the manufacturer's instructions. The ITS region (ITS1, 5.8S and ITS2) of ribosomal DNA (rDNA) was amplified with the forward primer NC5 (5′-GTAGGTGAACCTGCGGAAGGATCATT-3′) and the reverse primer NC2 (5′-TTAGTTTCTTTTCCTCCGCT-3′) [8]–[10]. Mitochondrial small subunit (SSU) of ribosomal RNA (rRNA) was amplified with the forward primer MH3 (5′-TTGTTCCAGAATAATCGGCTAGACTT-3′) and the reverse primer MH4 (5′-TCTACTTTACTACAACTTACTCC-3′). The PCR reaction was performed as previously described [17]. Each PCR product was digested by two restriction enzyme *Hha*I and *Hinf*I (Takara, Japan) and evaluated based on previous reports [8]–[10], [15]–[17]. To identify the hybrid genotype, the PCR products of the ITS and mitochondrial SSU regions were sequenced using the BigDye Terminator version 3.1 Cycle Sequencing kit (Applied Biosystems, USA) according to the manufacturer's instructions, and an ABI3100 automatic sequencer (Applied Biosystems, USA). Sequences were visualized using chromatograms, and were, manually corrected subsequently using the Finch TV software (Geospiza, USA).A BLAST search (National Center of Biotechnology Information public databases; http://blast.ncbi.nlm.nih.gov/Blast.cgi) was carried out to elucidate previously published sequences. Multiple sequence alignment was carried out using the Clustal W software [18].

### Identification of the penetration sites

Upper gastrointestinal endoscopy or colonoscopy was performed in all patients. All endoscopic examinations were conducted with an electronic scope (OLYMPUS or FUJINON, Tokyo, Japan). In all cases of gastric anisakiasis, we carefully examined each area infected by *Anisakis* and documented whether each larva was found in normal or atrophic mucosa. Normal mucosa was defined as homogeneously reddish, smooth, and regular arrangement of collecting venules, whereas atrophic mucosa was defined as yellowish-pale tissue, with transparent blood vessels without biopsies [19].

### Grading of gastric mucosa atrophy

The severity of gastric atrophy was determined based on the classification system of Kimura and Takemoto [20]. This classification divides the extent of atrophy into closed type (C-type) and open type (O-type). The C-type is subdivided into C1, C2, and C3, while the O-type is subdivided into O1, O2, and O3. The atrophic border is observed in the distal antrum in the C1 type, the lower and middle parts of the corpus in the C2 type, and the upper part of the corpus in the C3 type. In the O1-type, the atrophic border is within the cardiac lesion. In the O-2 type, it lies beyond the cardiac lesion to the anterior and posterior walls of the body. In the O-3 type, the endoscopic atrophic area is widely spread with border lying along the greater curvature. The area ratio of normal mucosa to atrophic mucosa was estimated using the figure adapted from S Ito (1996) [20] with imaging application (Image-J software, National Institutes of Health, Bethesda, Maryland, USA). To determine the impact of atrophy on the larvae penetration sites, C-0 (normal) and O-3 (almost completely atrophic mucosa) were excluded because the ratio varies depending on the selective site of invasion. Theoretical expectation of larvae in each condition of mucosa (from C-1 to O-2) was calculated as follows; the observation number of *Anisakis* larvae multiplied by the area ratio of normal mucosa to atrophic mucosa.

### Statistical analysis

Statistical analyses were performed using test for population proportion and Fisher's exact test (Statcel 3 software, OMS Publishing Inc., Saitama, Japan); *p* value of less than 0.05 was considered to be statistically significant.

### Ethics statement

The research was approved by the Ethical Committee of Tokyo Medical and Dental University, Kameda Medical Center, and Toukatsu Hospital, respectively. All cases were adult and all subjects were required to give written informed consent, and where such approval was not forthcoming, the subject concerned was excluded from the study.

## Results

### Characteristics of the patients

A total of 42 patients diagnosed with gastric anisakiasis and 2 patients with colonic anisakiasis were admitted in the three Middle Eastern Japan hospitals during the study period. The onset of symptoms was 1.4±1.1 days (range: 0–5 days) before admission to a hospital (Table 1). The sources of infection were raw or undercooked edible fish, including sardine (33%), mackerel (28%), and yellowtail snapper (21%). Interestingly, 11 cases were asymptomatic for anisakiasis. Four patients were admitted for a follow-up examination on gastric or colon cancer after endoscopic treatment, five patients we screened for gastric cancer by detailed endoscopic examination, and two patients were admitted for a follow-up examination on gastroesophageal reflux disease.

**Table 1:** Characteristics of the patients.

Number of patients	44
Gender (men/women)	16/28
Age, mean (SD)	54.5 (±15.6)
Antacid medicines (cases)	9
Source of infection (%)	
Sardines	33
Mackerel	28
Yellow tail	21
Horse mackerel	5
Flatfish	5
Rockfish	2
Trout	2
Squid	2
Tuna	2
Symptomatic/Asymptomatic (cases)	33/11
Onset of symptoms after eating, mean (SD) (day)	1.4 (±1.1)

### Morphological and genotyping of *Anisakis* larvae

**Figure 1:**
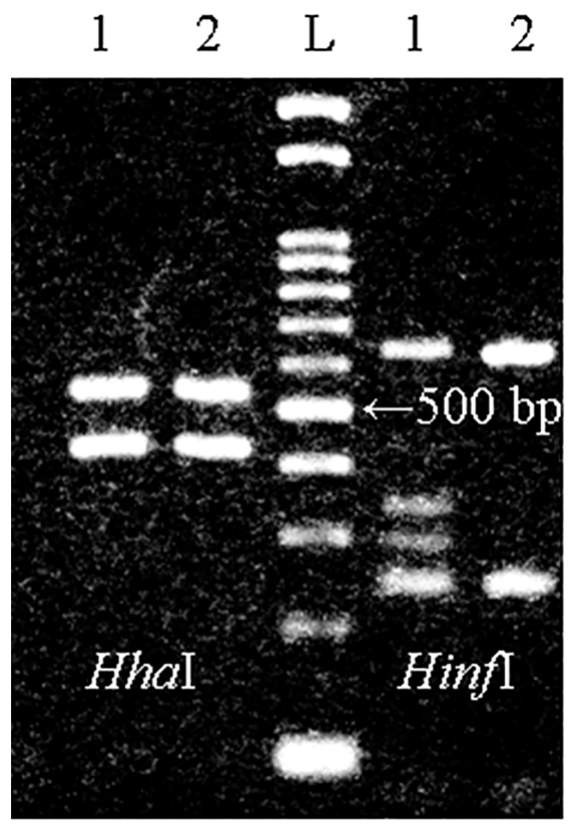
RFLP pattern of the ITS region under the restriction enzymes (*Hha*I and *Hinf*I). Restriction with *Hha*I produced two fragments of 550 bp and 430 bp in Hybrid genotype and *A. simplex* s. s.. *Hinf*I produced four fragments of 620 bp, 370 bp, 300 bp, and 250 bp in Hybrid genotype, whereas produced two fragments of 620 bp and 250 bp in *A. simplex* s. s.. Lanes: 1, Hybrid genotype; 2, *A. simplex* s. s.; L, 100-bp ladder.

In total, 55 larvae were removed by gastrointestinal endoscopy and 2 larvae by colonoscopy. Macroscopic examination revealed that all larvae belonged to the genus *Anisakis* sp. (Table 2). No *Pseudoterranova* type lava was found. Fifty-six larvae were identified at the 3^rd^ larval stage, and one larva exhibited the characteristics of the 4^th^ larval stage, with shedding of part of its outer cuticle and absence of mucron and boring tooth, as reported previously [6], [15]. There were no morphological differences between the symptomatic isolates and asymptomatic isolates. The RFLP profiles obtained by digestion of the ITS region (*Hha*I and *Hinf*I) showed that 99% of the larvae were A. *simplex* s.s. (550–430 and 620–250 bp), and one larva had a hybrid genotype (550–430 bp and 620–370–300–250 bp) (Figure 1). This observation was consistent with a hybrid genotype as reported previously [17]. To confirm the diagnosis, the ITS region and mitochondrial SSU regions were further examined by sequence analysis. The sequencing of the ITS region supported the presence of heterozygotes (C/T) at position 240 and 256. Mitochondrial SSU alignment revealed that the sequence of the hybrid was consistent with that of *A. simplex* s.s, but different from that of *A. pegreffii* at positions 30, 32, and 429 (see Table 3), which indicated the female parent of hybrid was *A. simplex* s.s..

**Table 2:** Characteristics of *Anisakis* larvae.

Stage of larvae (cases)	57
Stage III	56
Stage IV	1
Species (cases)	57
*A. simplex* s. s.	56
Hybrid genotype	1
Location of larvae (cases)	57
Stomach*	54
Duodenum	1
Colon	2
Number of cases in multiple infection (2–4 larvae)	7
Stomach	7
Colon	none

*52% was in the gastric body, and most of them in the greater curvature.

**Table 3:** Alignment of ITS and mitochondrial SSU regions.

Species of *Anisakis* larvae	Accession number	Position of ITS region		Position of mitochondrial SSU		
		240	256	30	32	429
*A. simplex* s.s.	AY826723/AY994157.1	T	T	C	G	A
*A. pegreffii*	AY826720/FJ810307.1	C	C	T	A	G
Clinical isolate	AB831878/AB894874	C/T	C/T	C	G	A

### Correlation between mucosal status on larval penetration

For this analysis, the larvae from patients who were prescribed antacid medicines were excluded because they may affect the morphology of the gastric mucosa. Accordingly, a total of 45 larvae from 35 patients were included. First, we identified a statistically significant correlation between clinical symptoms and penetration site (p = 0.014; Table 4). Patients with infected normal mucosa were more likely to exhibit symptoms (odds ratio, 6.96; 95% confidence interval, 1.52–31.8) than those with infected atrophic mucosa. Also, we tested whether the larvae preferentially infected normal or atrophic mucosa. Statistically significant correlation was found between the penetration sites and normal mucosa by test for population proportion (p = 0.005) (Table 5). Thus, we suggested that *A. simplex* s.s. tends to penetrate normal mucosa than atrophic area, regardless of the extent of background atrophic gastritis.

**Table 4:** Correlation between penetration site and symptoms.

	Penetration site in stomach	
	Atrophic mucosa	Normal mucosa
Symptomatic	5	29
Asymptomatic	6	5

**Table 5:** Correlation between penetrating site and mucosa background in the stomach.

	Surfece area		Observation number of larvae			Theoretical number of larvae		
Atrophic border	Normal	Atrophic	Normal	Atrophic	Total	Normal	Atrophic	Total
C1	0.83	0.17	5	0	5	4.15	0.85	5
C2	0.65	0.35	5	0	5	3.25	1.75	5
C3	0.44	0.56	3	1	4	1.76	2.24	4
O1	0.17	0.83	2	3	5	0.85	4.15	5
O2	0.1	0.9	2	2	4	0.4	3.6	4

## Discussion

Anisakiasis is a major issue in Japan due to the nutritional habits of the general population. The medical record of the patients included in this study revealed that they consume a large variety of raw or undercooked edible fishes as sashimi or sushi. Because this habit is deeply rooted in the culture of the country, the current focus is to improve the efficiency of endoscopic removal of the larvae implanted in the gastrointestinal wall. The present study identified the genotype of the larvae affecting the Middle Eastern Japanese population, and offers invaluable information on the distribution of the larvae in the gastric mucosa.

In Southern and Northern Japan, the major etiological agent of human anisakiasis was identified as *A. simplex* s.s. [10]. The present study identified the same parasite in 99% of the larvae isolated from Middle Eastern Japanese patients with gastric anisakiasis. Interestingly, 1 larva exhibited a hybrid genotype, which is the first report for human anisakiasis. The larva with the hybrid genotype was collected from a patient who ate trout and was infected with three larvae by gastrointestinal endoscopy; this patient had abdominal pain. The existence of a hybrid genotype was explained as the result of natural interspecies hybridization between *A. simplex* s.s. and *A. pegreffii* [17], [21], [22]. In the previous study, hybrid genotypes were detected in *Blue whiting*, *Scomber japonicas, and Scomber scombrus* [21], [22].We hypothesized that the hybrid genotype could parasite in various marine fish. There was no morphological difference between *A. simplex* s.s. and the hybrid genotype. Moreover, we didn`t find the pathogenic characteristics between *A. simplex* s.s. and the hybrid genotype. Further studies are required to characterize the hybrid genotype.

These data suggest that *A. simplex* s.s. is the causative agent of anisakiasis everywhere in Japan. Incidentally, *A. simplex* s.s. larvae was reported to penetrate muscle tissue more easily than *A. pegreffii* larvae in edible fish [23], [24]. Therefore, the predominance of gastric infection by *A. simplex* s.s. does not necessarily reflect the relative abundance of these parasites in the ocean.

The present study also addressed the challenging task of locating and removing all *Anisakis* larvae lodged in the gastric mucosa of a patient. Close endoscopic examination of the infected areas revealed that the larvae penetrated normal mucosa significantly more often than the atrophic area. A recent study suggests that regional pH differences may be responsible for this behavior. They showed that *A. simplex* s.s. larvae penetrated agar gel more efficiently at lower pH, and survived longer in gastric juice than *A. pegreffii* larvae [25]. These data are consistent with the lower pH measured in normal gastric mucosa than in atrophic gastric mucosa [14]. A recent report suggests that the use of narrow band imaging improves the detection of *Anisakis* larvae in the gastrointestinal wall [26]. Therefore, when endoscopic examination for anisakiasis is conducted, it is very important to examine normal mucosa very carefully using narrow band imaging.

We also report the puzzling observation that patients with *Anisakis* larvae lodged in normal mucosa exhibited clinical symptoms more frequently than those infected in atrophic area. This is an alarming observation because patients already facing digestive complications causing extensive mucosal atrophy may not manifest the typical clinical symptoms. These patients may have undiagnosed anisakiasis for years and suffer from unexplained complications. For example, a recent case-control study showed that prior *Anisakis* infection is an independent risk factor for upper gastrointestinal bleeding, and amplifies 3-fold the adverse effects of non-steroidal anti-inflammatory drugs (NSAIDs) on the joints [27]. Therefore, patients who have received treatment for anisakiasis should be monitored closely when they are prescribed NSAIDs for a chronic condition.

It is still unclear why patients with *Anisakis* larvae infecting their normal gastric mucosa are more likely to exhibit clinical symptoms than those with atrophic mucosa infections. Several reports mentioned that symptomatic anisakiasis is caused by an allergic response [28], [29]. Accordingly, clinicians noted the efficacy of corticosteroids therapy for gastric and intestinal anisakiasis [30]. Since this is a retrospective study, anti-*Anisakis* IgE antibody was not determined. Future studies comparing serum IgE levels between symptomatic and asymptomatic patients may shed some light on the mechanisms of anisakiasis.

Thus, after treatment of anisakiasis, it may be necessary to follow-up when the patients take NSAIDs. In conclusion, the present study showed that *A. simplex* s.s. is the predominant etiological agent for anisakiasis in Japan, and the larvae preferentially penetrate normal gastric mucosa. Careful endoscopic examination of normal mucosa, particularly in the greater curvature of the gastric body, will improve detection of the larvae and treatment efficiency. Finally, since patients with widespread atrophic mucosa may not manifest the clinical symptoms of anisakiasis, routine endoscopic screening could potentially avoid unexplainable complications and adverse reactions to common medications.
